# Outcomes of Near-Infrared Photoimmunotherapy for Head and Neck Cancer: A Single-Center Retrospective Study

**DOI:** 10.3390/cancers18030350

**Published:** 2026-01-23

**Authors:** Hiroaki Tahara, Tsutomu Ueda, Takayoshi Hattori, Minoru Hattori, Yuki Sato, Nobuyuki Chikuie, Takayuki Taruya, Takao Hamamoto, Takashi Ishino, Sachio Takeno

**Affiliations:** 1 Department of Otorhinolaryngology, Head and Neck Surgery, Hiroshima University, Kasumi 1-2-3 Minami-ku, Hiroshima 734-8551, Japan; ht0115@hiroshima-u.ac.jp (H.T.); hatt@hiroshima-u.ac.jp (T.H.); sato0123@hiroshima-u.ac.jp (Y.S.); housejak@hiroshima-u.ac.jp (N.C.); ttaruya@hiroshima-u.ac.jp (T.T.); takao0320@hiroshima-u.ac.jp (T.H.); tishino@hiroshima-u.ac.jp (T.I.); takeno@hiroshima-u.ac.jp (S.T.); 2 Center for Medical Education, Institute of Biomedical & Health Sciences, Hiroshima University, Hiroshima 734-8551, Japan; m-hattori@hiroshima-u.ac.jp

**Keywords:** near-infrared photoimmunotherapy, head and neck cancer, cetuximab sarotalocan sodium, unresectable

## Abstract

Background/Objectives: Near-infrared photoimmunotherapy (NIR-PIT) represents a treatment approach for patients with locally advanced or recurrent head and neck cancers who are unsuitable for surgery post-standard therapy. Since its introduction in Japan in January 2021, NIR-PIT has been available exclusively under the national health insurance system, resulting in limited real-world clinical practice data. This study evaluated the association between NIR-PIT and overall survival (OS) in clinical practice. Methods: This single-center retrospective study included 45 patients with head and neck cancer who were not amenable to surgical resection owing to advanced local disease or regional recurrence without distant metastasis and who underwent NIR-PIT or systemic pharmacotherapy between January 2021 and April 2025. The primary endpoint was OS. Results: Twenty-two and 23 patients received NIR-PIT and pharmacotherapy, respectively. In the NIR-PIT group, irradiation was delivered to primary tumors in 20 patients, cervical lymph node lesions in one, and both primary and lymph node lesions in one. The median OS was 35 and 8 months, with median follow-up of 40 and 49 months in the NIR-PIT and pharmacotherapy groups, respectively. Among the NIR-PIT-eligible patients, 22 and 10 were treated with NIR-PIT and pharmacotherapy, respectively. The median OS was 35 and 8 months, with median follow-up of 40 and 24 months in the NIR-PIT and pharmacotherapy groups, respectively. Conclusions: NIR-PIT was independently associated with improved OS in patients with unresectable recurrent or metastatic head and neck cancer without distant metastasis. Prospective multicenter studies are warranted to validate these findings.

## Simple Summary

Near-infrared photoimmunotherapy (NIR-PIT) is a novel treatment option for patients with unresectable recurrent or metastatic head and neck cancer without distant metastasis. We retrospectively compared outcomes between patients treated with NIR-PIT and those treated with systemic pharmacotherapy during the same period. Disease control, inflammatory markers reflecting systemic immune status, and NIR-PIT use were associated with overall survival. Among patients who were technically eligible for NIR-PIT, disease control and NIR-PIT administration remained associated with survival. In this preliminary, hypothesis-generating study, our findings suggest that NIR-PIT contributes to improved survival in carefully selected patients.

## 1. Introduction

Head and neck cancer arises from multiple anatomical sites, including the oral cavity, pharynx, and larynx, and collectively account for a large number of cancer diagnoses worldwide, with nearly 900,000 new cases annually [1,2]. In Japan, the annual incidence and mortality of head and neck cancer are projected to reach 27,400 and 9600, respectively [3]. Surgical treatment for recurrent and metastatic head and neck cancer in patients who have previously undergone radiation or surgery has a high risk of severe complications [4,5]. Consequently, systemic therapies, such as chemotherapy, molecular targeted agents, and immune checkpoint inhibitors (ICIs), which have rapidly developed in recent years, are commonly used [6,7,8]. However, these treatments demonstrate low cure rates even for localized diseases [9]. Therefore, near-infrared photoimmunotherapy (NIR-PIT) has emerged as a new modality for achieving local control of head and neck cancer [10,11].

Recent preclinical studies have demonstrated that tumor microenvironment–responsive nanoplatforms can enhance the efficacy of photodynamic therapy through multimodal therapeutic strategies [12].

NIR-PIT biologically and clinically differs from conventional photodynamic therapy (PDT) in the following ways. Biologically, conventional PDT induces cytotoxicity primarily through the generation of reactive oxygen species when a photosensitizer is activated by light, leading to oxidative damage and apoptosis or necrosis of cancer cells. Clinically, it lacks high specificity because photosensitizers tend to accumulate in tumors and normal tissues, risking collateral damage.

NIR-PIT employs a laser-based irradiation system following the administration of cetuximab sarotalocan sodium [10]. This agent consists of an epidermal growth factor receptor (EGFR)-targeting monoclonal antibody conjugated to the near-infrared photosensitizer IR700. Selective cytotoxic effects are induced in EGFR-expressing tumor cells upon exposure to near-infrared light at 690 nm. Its use in NIR-PIT provides deeper penetration into biological tissues than the visible or shorter-wavelength light used in conventional PDT, which is limited by absorption and scattering, making NIR-PIT more suitable for treating deeper tumors [13,14].

NIR-PIT is currently performed in Japan under insurance coverage for unresectable, locally advanced, or recurrent head and neck cancer after standard therapies such as surgery and radiation. It enables effective local tumor control while minimizing systemic toxicity compared with systemic pharmacotherapy and preserving surrounding normal tissue through this spatial selectivity.

Since NIR-PIT became available in Japan in January 2021, most published reports have focused on single cases or small case series [15,16,17,18]. Four years after its approval for insurance coverage, reports with several dozen case series from single- and multi-center studies have emerged [19,20,21]. Therefore, this study aimed to assess the efficacy and safety of NIR-PIT in more than 20 patients with unresectable, locally advanced, or locally recurrent head and neck cancer without distant metastasis who underwent NIR-PIT. Overall survival (OS) was compared with that of a contemporaneous cohort treated with systemic pharmacotherapy alone during the same period.

## 2. Materials and Methods

### 2.1. Study Design and Population

This single-center, retrospective, observational study was conducted at the Department of Otorhinolaryngology and Head and Neck Surgery of Hiroshima University Hospital. Patients with unresectable, locally advanced, or locoregionally recurrent head and neck squamous cell carcinoma (SCC) without distant metastasis were identified from electronic medical records between January 2021 and April 2025. The inclusion criteria were age ≥ 18 years, histopathologically confirmed SCC or EGFR expression, Eastern Cooperative Oncology Group performance status (ECOG PS) 0–2, completion of standard therapy (radiotherapy and/or surgery), and absence of distant metastases. Patients with known hypersensitivity to cetuximab sarotalocan sodium, active autoimmune disease requiring systemic immunosuppression, carcinoma of unknown primary origin, external auditory canal carcinoma, or concurrent participation in interventional clinical trials were excluded. During the study period, 48 patients met the inclusion criteria, of whom 45 were included in the analysis after three were excluded based on the predefined exclusion criteria. Patients were stratified into two cohorts based on treatment allocation determined by a multidisciplinary team evaluation. The NIR-PIT group (*n* = 22) included patients without carotid artery encasement or invasion, and with tumors located in anatomically accessible sites amenable to laser delivery. Conversely, the pharmacotherapy group (*n* = 23) consisted of patients who were treated with pharmacotherapy alone during the same period, primarily because NIR-PIT was not selected owing to technical limitations in laser delivery for deeply located lesions or patient preference. Among them, 13 cases were due to technical difficulties in performing NIR-PIT, while 10, although eligible for NIR-PIT, underwent systemic therapy based on patients’ preferences, such as the desire for organ preservation.

Patient demographics, tumor characteristics, pre-treatment laboratory parameters (neutrophil-to-lymphocyte ratio [NLR], platelet-to-lymphocyte ratio [PLR], and modified Glasgow Prognostic Score [mGPS]), and clinical outcomes were retrospectively extracted from medical records. Post-treatment NLR and PLR in the NIR-PIT group were also retrospectively extracted from medical records.

### 2.2. Treatment with Near-Infrared Photoimmunotherapy (NIR-PIT)

Treatment consisted of an intravenous infusion of cetuximab sarotalocan sodium (Akalux^®^, Rakuten Medical, Tokyo, Japan) at a dose of 640 mg/m^2^, administered over at least 2 h. Laser irradiation was performed 20–28 h after completing the infusion under general anesthesia in an operating room. Cetuximab sarotalocan sodium is a conjugate of cetuximab—a chimeric IgG1 monoclonal antibody targeting EGFR—and IRDye700DX (IR700)—a near-infrared photosensitizer. Preparation and handling of the infusion solution were performed under reduced illuminance (≤120 lx) since IR700 is light-sensitive, and the infusion bag was covered with a light-shielding film.

**Figure 1:**
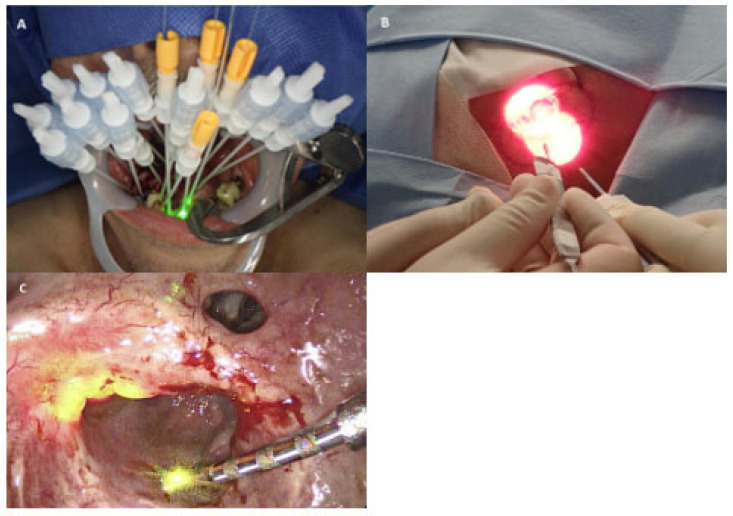
Three types of diffusers used in NIR-PIT. (**A**) Needle catheters used to insert cylindrical diffusers into the tumor, with the guide light visible as a green glow. (**B**) Frontal diffusers used to irradiate a tumor located posterior to the ear. (**C**) A side-fire diffuser used to irradiate a tumor in the soft palate. NIR-PIT, near-infrared photoimmunotherapy.

NIR light at a wavelength of 690 nm was delivered using a semiconductor laser system (BioBlade^®^ laser, Rakuten Medical, Tokyo, Japan). External irradiation was performed for superficial lesions at an output density of 150 mW/cm^2^ with a total energy of 50 J/cm^2^. For deep-seated lesions, interstitial irradiation was performed by inserting a diffuser into the tumor, delivering an output density of 400 mW/cm and a total energy of 100 J/cm per diffuser length. Illumination was performed using frontal, side-fire, or cylindrical diffuser probes, selected according to tumor location, depth, and anatomical constraints. Frontal and side-fire diffusers were used for superficial lesions and generated circular irradiation fields with diameters of approximately 7–38 mm. In contrast, cylindrical diffusers were inserted through needle catheters to provide radial intratissue illumination with an effective radius of approximately 10 mm (corresponding to a 20 mm diameter). The illumination field was set with a safety margin of 5–10 mm beyond the visible tumor margin, and each irradiation time was approximately 5 min 33 s for external irradiation and 4 min 10 s for interstitial irradiation; multiple sessions were performed as needed to cover the entire lesion. Representative photographs showing irradiation using frontal, side-firing, and cylindrical diffuser probes are presented in Figure 1.

**Figure 2:**
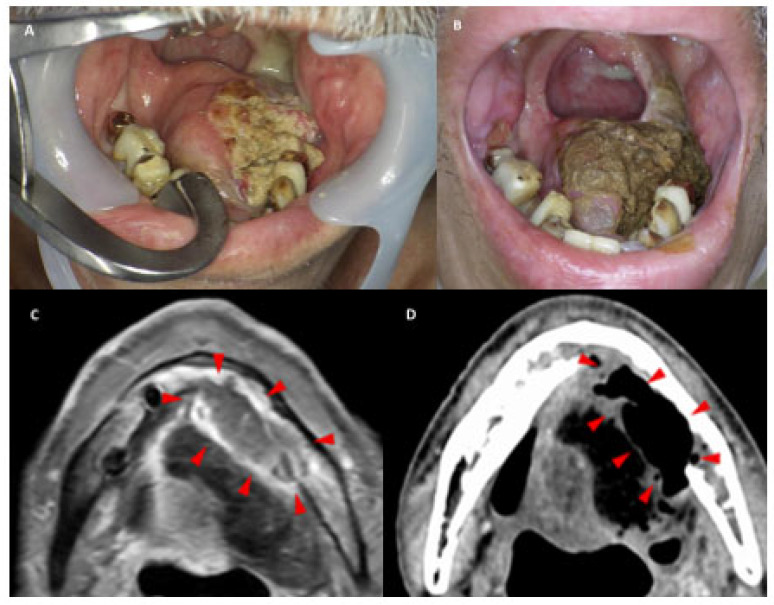
A representative case of recurrence after tongue cancer reconstruction surgery. (**A**) Tumor recurrence observed on the lateral aspect of the mandible, medial to the reconstructed tongue. (**B**) Tumor necrosis with marked tumor shrinkage observed 1 month post-treatment. (**C**) Axial contrast-enhanced T1-weighted MRI showing the extent of the tumor (arrowheads). (**D**) Axial contrast-enhanced CT scan obtained 1 month post-treatment shows a marked reduction in the tumor (arrowheads). MRI, magnetic resonance imaging; CT, computed tomography.

Up to four NIR-PIT cycles were permitted, with a minimum interval of 4 weeks between cycles, in accordance with previous clinical trial protocols [22]. Treatment continuation was determined based on clinical response, lesion accessibility, and patient tolerance. However, treatment was discontinued upon achievement of a complete response (CR) and was terminated in cases of disease progression or severe adverse events. Retreatment was considered in cases of residual or recurrent disease, provided that the lesion remained accessible to laser illumination and the patient’s general condition was adequate. All procedures were performed under general anesthesia. Orotracheal or nasotracheal intubation was selected for airway management according to the irradiation site. Tracheostomy was concurrently performed when prolonged laryngeal edema was anticipated. Sedation and respiratory management were tailored to the tumor site and patient’s status. Figure 2 presents representative local clinical photographs and imaging studies obtained pre- and post-treatment, demonstrating the therapeutic effects of NIR-PIT.

### 2.3. Outcomes and Assessments

The primary endpoint was to investigate factors associated with OS in patients with unresectable recurrent or metastatic head and neck cancer without distant metastasis. OS was measured from the initiation of therapy for recurrent or metastatic lesions after completing standard therapy, which refers to prior definitive treatments, such as surgery or chemoradiotherapy, until all-cause mortality. Multivariate analysis was performed to identify factors associated with OS.

Secondary endpoints included treatment effectiveness, best overall response (BOR), objective response rate (ORR), and disease control rate (DCR).

The patients included in this analysis had recurrent or metastatic lesions without distant metastases. All existing lesions were treated. Accordingly, treatment response was evaluated using the Response Evaluation Criteria in Solid Tumors version 1.1 [23].

NIR-PIT was administered to the primary tumor and/or cervical lymph nodes when technically feasible. Therapeutic response to NIR-PIT was evaluated 1 month after each treatment cycle, which is clinically appropriate given the minimum 4-week interval required between cycles for retreatment decision-making. In contrast, therapeutic response to systemic pharmacotherapy was evaluated every 2–3 months using computed tomography, magnetic resonance imaging, and positron emission tomography.

### 2.4. Data Analysis

Continuous and categorical variables are reported as medians (ranges) and proportions and/or percentages, respectively. Categorical data were analyzed using Fisher’s exact test, whereas continuous data between groups were compared using the Mann–Whitney U test. The Kaplan–Meier method and log-rank test were used to compare the OS. Hazard ratios (HRs) were calculated using the cumulative survival function. Univariate and multivariate analyses of OS were performed using Cox proportional hazards models. Multivariate Cox regression analysis was performed on significant variables in the univariate analysis. HR and corresponding 95% confidence intervals (CIs) were evaluated. A univariate logistic regression analysis was initially performed to investigate the association between these variables and OS, providing an odds ratio (95% CI) for each variable. To determine whether the markers exhibited independent associations with OS, we performed multivariate logistic regression analyses on significant variables in the univariate analysis to obtain the adjusted odds ratio (95% CI). All statistical analyses were performed using IBM SPSS Statistics for Windows, version 27 (IBM Corp., Armonk, NY, USA), with statistical significance set at *p* < 0.05.

### 2.5. Ethical Considerations

This study’s protocol was approved by the Ethical Review Board of Hiroshima University Hospital (approval number: E-2039), conducted in accordance with the principles of the Declaration of Helsinki, and posted at our institution. All patients were given the choice to opt out of the study.

## 3. Results

### 3.1. Characteristics of the Patients

Forty-five patients underwent treatment during the study period. Of these, 22 and 23 received NIR-PIT (NIR-PIT group) and systemic pharmacotherapy without NIR-PIT (pharmacotherapy group), respectively. Table 1 presents a summary of baseline patient characteristics. In the NIR-PIT group, the median age was 68 (range: 50–81) years, with 17 males and 5 females. The median age was 70 (range: 51–93) years in the pharmacotherapy group, with 22 males and 1 female. Histological analysis revealed that 21 patients (95.5%) in the NIR-PIT group had SCC, while the remaining patients had salivary duct carcinoma with EGFR expression. All 23 patients in the pharmacotherapy group had pathologically confirmed SCC. The primary tumor sites in the NIR-PIT group were the oral cavity (*n* = 5), oropharynx (*n* = 7; p16-positive, *n* = 1; p16-negative, *n* = 6), nasopharynx (*n* = 3), hypopharynx (*n* = 2), larynx (*n* = 1), nasal sinus (*n* = 3), and parotid gland (*n* = 1). In the pharmacotherapy group, they were the oral cavity (*n* = 7), oropharynx (*n* = 4; p16-positive, *n* = 1; p16-negative, *n* = 3), nasopharynx (*n* = 1), hypopharynx (*n* = 5), larynx (*n* = 2), and nasal sinus (*n* = 3). Recurrent TNM (rTNM) staging was assessed according to the American Joint Committee on Cancer (AJCC) TNM staging system, 8th edition [24]. All patients were classified as rM0. In the NIR-PIT group, rT-stages were rT0 (*n* = 1), rT1 (*n* = 1), rT2 (*n* = 9), rT3 (*n* = 4), and rT4 (*n* = 7), while rN-stages were rN0 (*n* = 20), rN1 (*n* = 0), rN2 (*n* = 0), and rN3 (*n* = 2). In the pharmacotherapy group, rT-stages were rT0 (*n* = 7), rT1 (*n* = 2), rT2 (*n* = 1), rT3 (*n* = 2), and rT4 (*n* = 11), whereas rN-stages were rN0 (*n* = 16), rN1 (*n* = 1), rN2 (*n* = 1), and rN3 (*n* = 5). Regarding target lesions, the pharmacotherapy group tended to have more cervical lymph nodes as target lesions than the NIR-PIT group. Since NIR-PIT cannot be performed in the presence of carotid artery invasion, no cases were included in the NIR-PIT group; however, four cases were identified in the pharmacotherapy group. In the NIR-PIT group, prior treatments for the primary site included surgery (*n* = 4), radiotherapy (*n* = 8), and a combination of surgery and radiotherapy (*n* = 10). In the pharmacotherapy group, prior treatments for the primary site included surgery (*n* = 6), radiotherapy (*n* = 11), and a combination of surgery and radiotherapy (*n* = 6). Pre-treatment blood biochemical parameters, such as NLR, PLR, and mGPS, were also evaluated, with no statistically significant differences found between the two groups.

### 3.2. Treatment Effectiveness

Table 2 shows the BOR during the study period for the NIR-PIT and pharmacotherapy groups. In the NIR-PIT group, CR, partial response (PR), stable disease (SD), and progressive disease (PD) were observed in 7 (31.8%), 11 (50.0%), 2 (9.1%), and 2 (9.1%) cases, respectively. The ORR was 81.8% and DCR was 90.9%. In the pharmacotherapy group, CR, PR, SD, and PD were observed in 1 (4.3%), 8 (34.8%), 8 (34.8%), and 6 (26.1%) cases, respectively. The ORR was 39.1%, and the DCR was 73.9%. Treatment response was assessed at 1 month post-treatment in the NIR-PIT group according to the protocol, whereas evaluations were performed every 2–3 months in the pharmacotherapy group.

### 3.3. Treatment Cycles of NIR-PIT

The number of NIR-PIT treatment cycles administered is described below. Six patients received one cycle, seven received two cycles (three of which were ongoing), five received three cycles (one of which was ongoing), and four received four cycles. Reasons for discontinuing treatment included PD, CR (all target lesions were considered to have achieved a CR following NIR-PIT), drug-induced pneumonia, exacerbation of other diseases, pain, and a lack of desire.

### 3.4. Safety

Table 3 presents the adverse events associated with NIR-PIT. They were evaluated according to the Common Terminology Criteria for Adverse Events version 5.0 [25]. Pain was observed in all patients and was graded as G1 or G2. Laryngeal edema occurred in 59% of patients and was mostly graded as G2 or lower; however, one patient had G4 laryngeal edema requiring emergency tracheostomy. Drug-induced pneumonitis, pharyngocutaneous fistula, and sepsis (Lemierre syndrome) were each observed in one patient. No cases of photosensitivity, which is a characteristic adverse event associated with NIR-PIT, were observed. No grade 5 adverse events occurred.

### 3.5. Factors Associated with OS

The factors associated with OS were evaluated. Prognostic factors associated with OS in the entire group of patients treated with NIR-PIT and pharmacotherapy were initially explored. In the univariate analysis, NLR, PLR, mGPS, treatment modality (NIR-PIT or pharmacotherapy), and DCR were identified as significant factors. Subsequent multivariate analysis demonstrated that NLR, PLR, treatment modality, and DCR were independently associated with OS. These results are presented in Supplementary Tables S1 and S2.

Given that treatment modality emerged as an independent prognostic factor, additional analyses were performed comparing patients in the NIR-PIT group and those in the pharmacotherapy group who were considered eligible for NIR-PIT.

Univariate analysis was performed to determine the background characteristics of each patient. Significant differences were observed in the ECOG PS (*p* < 0.001), primary site (*p* < 0.001), treatment modality (*p* = 0.003), and DCR (*p* = 0.01) (Table 4). Multivariate analysis of these parameters showed significant differences in treatment modality (*p* = 0.018) and DCR (*p* = 0.03), indicating that they were independent factors associated with OS (Table 5).

Although ICI history is a clinically relevant factor, it was not included in the multivariate analyses because all patients with prior ICI therapy belonged to the NIR-PIT group, making its effect statistically non-identifiable due to perfect collinearity with treatment assignment.

### 3.6. OS According to Treatment Modality

Supplementary Figure S1 shows the Kaplan–Meier curves for the entire group. OS was significantly prolonged in the NIR-PIT group compared with the pharmacotherapy group. The median OS was 35 and 8 months in the NIR-PIT and pharmacotherapy groups, respectively (log-rank *p* = 0.001). Moreover, the median follow-up was 40 and 49 months in the NIR-PIT and pharmacotherapy groups, respectively.

**Figure 3:**
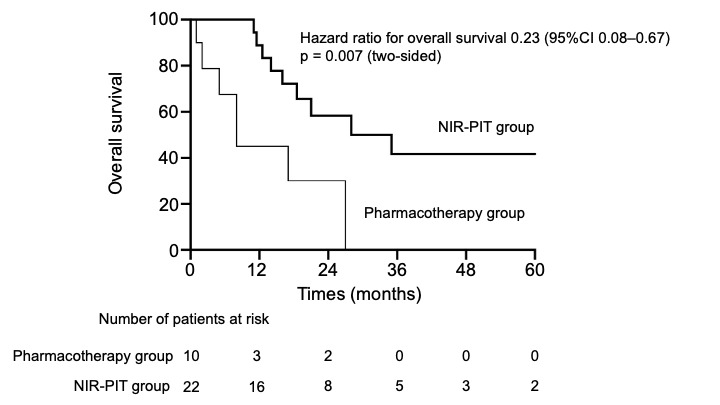
Kaplan–Meier curves of overall survival in patients eligible for NIR-PIT. NIR-PIT, near-infrared photoimmunotherapy.

Figure 3 presents the Kaplan–Meier curves of OS in patients in the NIR-PIT and pharmacotherapy groups. OS was significantly prolonged in the NIR-PIT group compared with the pharmacotherapy group. The median OS was 35 and 8 months in the NIR-PIT and pharmacotherapy groups, respectively (log-rank *p* = 0.007). Additionally, the median follow-up was 40 and 24 months in the NIR-PIT and pharmacotherapy groups, respectively. This result demonstrates a statistically and clinically meaningful survival benefit in the NIR-PIT group.

A significant difference in OS was observed between patients who underwent NIR-PIT and those who did not. To examine OS in individual cases in the NIR-PIT and pharmacotherapy groups, we created swimmer plots for the OS of each group and analyzed them.

**Figure 4:**
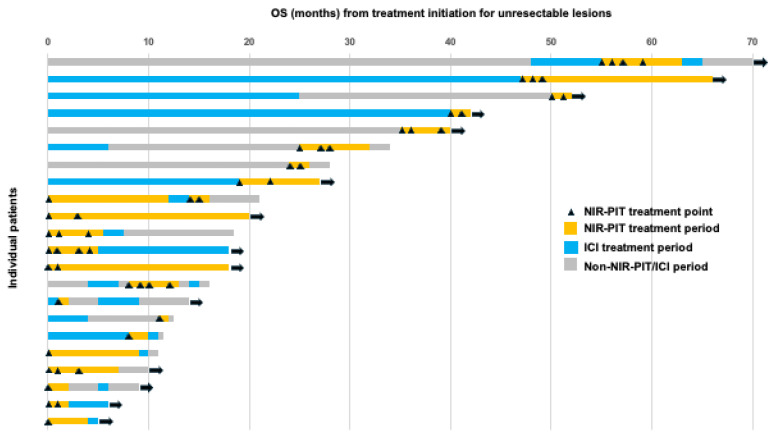
Swimmer plot of the OS of patients who underwent NIR-PIT. Each bar represents a single patient, with the length of the bar corresponding to OS. The right-pointing arrow indicates that the patient is ongoing. OS, overall survival; NIR-PIT, near-infrared photoimmunotherapy; ICI, immune checkpoint inhibitor.

Figure 4 shows the OS from treatment initiation for unresectable lesions in the NIR-PIT cohort. The longest OS was 70 months, followed by 66 months, with the latter patient remaining alive without disease post-treatment for nasopharyngeal carcinoma. Among the 22 patients, 14 (63.6%) remained alive, and a relatively large proportion achieved long-term survival.

**Figure 5:**
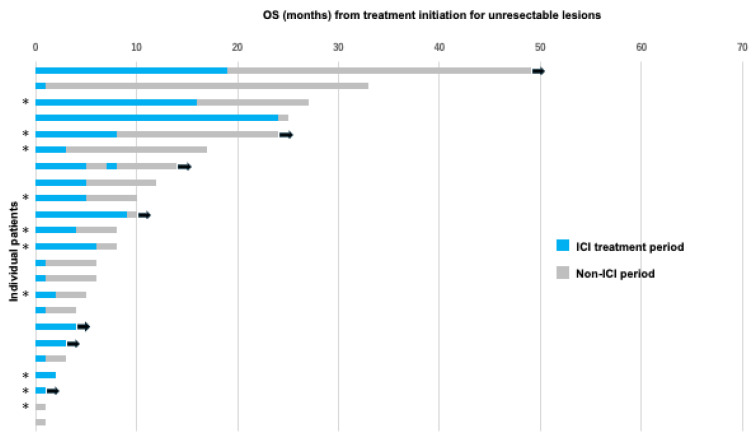
Swimmer plot of the OS of patients with Pharmacotherapy. Each bar represents a single patient, with the length of the bar corresponding to the OS. The right-pointing arrow indicates the patient’s current state. The cases marked with * were eligible for NIR-PIT, but pharmacotherapy was selected based on patient preference. OS, overall survival; NIR-PIT, near-infrared photoimmunotherapy; ICI, immune checkpoint inhibitor.

Figure 5 depicts the OS from treatment initiation for recurrent and metastatic lesions in the cohort treated with pharmacotherapy, primarily chemotherapy, without NIR-PIT. The longest OS was 49 months. ICIs were administered to 21 of the 23 patients (91.3%). Compared with the NIR-PIT cohort, only 7 of the 23 patients (30.4%) remained alive at the time of analysis.

We further examined the association of baseline and post-treatment changes in NLR and PLR with OS among patients who underwent NIR-PIT. Changes in baseline and post-treatment NLR showed a weak correlation with OS. The results are provided in Table 6.

## 4. Discussion

We conducted a comparative analysis of NIR-PIT and pharmacotherapy administered during the same period in patients with unresectable recurrent or metastatic head and neck cancer without distant metastasis. To our knowledge, this is the first study to directly compare NIR-PIT and pharmacotherapy with a primary focus on OS in this clinical setting.

In the analysis of irradiation sites, the pharmacotherapy group tended to have a greater number of cervical lymph nodes as target lesions than the NIR-PIT group. This difference is partially attributable to the inclusion of four patients with carotid artery invasion in the pharmacotherapy group. NIR-PIT is generally considered clinically contraindicated in cases of carotid artery invasion because of concerns regarding the risk of vascular rupture.

Regarding treatment effectiveness, the NIR-PIT group demonstrated more favorable outcomes than the Pharmacotherapy group in terms of BOR, ORR, and DCR. The DCR achieved with NIR-PIT at our institution was 90.9%, which is generally consistent with previous reports, including a DCR of 95.0% in 40 patients treated with NIR-PIT reported by Okamoto et al. [20], as well as an unconfirmed DCR of 80.0% reported in the phase 1/2 RM-1929 trial [22].

Nevertheless, these short-term response outcomes should be interpreted cautiously. Imaging assessments were performed monthly in the NIR-PIT group according to the treatment protocol, whereas patients in the pharmacotherapy group typically underwent imaging evaluations every 2–3 months. This difference in assessment frequency may have influenced response evaluation and potentially resulted in an overestimation of treatment effectiveness in the NIR-PIT group. Therefore, direct comparisons based solely on short-term response metrics have inherent limitations, and long-term follow-up focusing on survival outcomes is essential for a more accurate assessment of the comparative efficacy of these treatment modalities.

Regarding the safety of NIR-PIT, Okamoto et al. reported on quality of life (QOL) and treatment-related adverse events and found that severe adverse events were rare [19]. Consistent with these findings, most adverse events observed at our institution were mild to moderate, with grade 2 or lower pain and laryngeal edema being the most common, while severe adverse events were infrequent.

Treatment-related pain, which may adversely affect QOL, was managed at our institution using intraoperative nerve blocks with long-acting local anesthetics, postoperative patient-controlled intravenous analgesia, and non-steroidal anti-inflammatory drugs. Shibutani et al. demonstrated the effectiveness of opioid analgesics for pain control after NIR-PIT [26]. Accordingly, opioids may be considered in selected patients with severe or refractory pain to optimize postoperative QOL.

Although the number of patients was limited, several severe adverse events were observed in the NIR-PIT group, including grade 4 laryngeal edema, grade 3 fistula formation, and grade 4 sepsis (Lemierre syndrome). Emergency tracheostomy, gastrostomy, and intensive care unit management were required in these cases. Importantly, all patients recovered from these complications and were ultimately discharged home. Similar severe adverse events have been reported by other institutions [20,27,28].

While NIR-PIT can be associated with serious complications, it can be performed safely when appropriate patient selection is under careful monitoring and timely clinical management.

Multivariate analysis of factors associated with OS in patients with recurrent or metastatic head and neck cancer without distant metastasis revealed that NLR, PLR, treatment modality, and DCR were independent prognostic factors.

NLR and PLR have been reported as inflammation-based markers with prognostic significance in head and neck cancer [29,30,31]. Previous studies have demonstrated that elevated baseline or on-treatment NLR and PLR are associated with poorer survival outcomes. Our findings are consistent with these reports and suggest that systemic inflammatory status influences prognosis even in patients undergoing NIR-PIT or pharmacotherapy.

Regarding treatment modality, treatment with NIR-PIT remained independently associated with OS, with a median OS of 35 and 8 months in the NIR-PIT and pharmacotherapy groups, respectively. For reference, the RM-1929 phase I/II trial reported a median OS of 9.3 months [22]. However, direct comparison between these studies should be interpreted cautiously, as OS was defined differently. In the present study, OS was measured from treatment initiation, whereas it was measured from the initiation of NIR-PIT in the RM-1929 trial. The KEYNOTE-048 trial reported median OS values of 11.6 and 13.0 months for ICI monotherapy and combination therapy with chemotherapy, respectively [8]. When considered in this clinical context, the OS observed in our NIR-PIT cohort appears relatively prolonged, although cross-trial comparisons remain inherently limited.

Because the pharmacotherapy group included patients for whom NIR-PIT was technically infeasible, an additional analysis restricted to patients who were technically eligible for NIR-PIT was performed. Treatment modality remained independently associated with OS even in this restricted cohort, supporting the hypothesis that NIR-PIT may contribute to improved survival outcomes in selected patients. Nevertheless, residual confounding cannot be excluded, and these findings should be regarded as hypothesis-generating rather than confirmatory.

As shown in the swimmer plot in Figure 4, programmed death-1 (PD-1) inhibitors were frequently selected as subsequent therapy, including for patients who exhibited PD despite undergoing NIR-PIT. Previous case reports have described remarkable responses to ICI administered after NIR-PIT [32], as well as cases in which patients who showed PD during prior ICI therapy achieved a dramatic response when rechallenged with ICI after NIR-PIT [33]. Additionally, previous clinical observations have shown that NIR-PIT induces systemic immune-related changes, which may provide a biological rationale for combination or sequential treatment strategies involving PD-1 inhibitors [34]. In this study, eight patients received ICI therapy after NIR-PIT as a subsequent treatment. The BOR for ICI was CR in one patient, SD in one, PD in five, and not evaluable in one. Since disease progression has been observed in many cases, further accumulation and analysis of cases are needed to determine whether ICI is an appropriate post-NIR-PIT option. In contrast, patients who achieved CR maintained this response even after the discontinuation of ICI upon patient request. Similar to previous case reports [32,33], our study included a case in which ICI demonstrated remarkable efficacy after PIT. Currently, a phase 1b/2 clinical trial combining NIR-PIT with ICIs is ongoing [35]. Synergistic antitumor effects have been reported when NIR-PIT is combined with ICI in preclinical mouse models, resulting in enhanced tumor-specific immune responses and improved tumor control [36,37]. Depending on the results of this trial, the combination therapy of NIR-PIT and systemic drug treatments may hold promise as a future treatment strategy.

Because NIR-PIT is designed to induce immunogenic cell death, we evaluated immune activation by analyzing changes in NLR and PLR following irradiation. Consequently, changes in NLR and PLR before and after the first NIR-PIT session showed a weak but observable correlation with OS. These findings should be interpreted cautiously due to the exploratory nature of the analysis and the limited sample size. Moreover, these findings further suggest that combining NIR-PIT with ICIs represents a potentially effective therapeutic strategy. Therefore, the optimal sequencing and patient selection for ICI after NIR-PIT remain to be clarified in prospective trials.

This study has some limitations. First, it was a single-center retrospective study with a limited sample size. Second, baseline characteristics differed between treatment groups, and selection bias could not be fully avoided. Accordingly, any observed difference in OS cannot be conclusively attributed to a direct effect of NIR-PIT. Third, the heterogeneity in tumor location, tumor burden, and prior treatment history limited stratified analyses. Finally, follow-up duration was relatively short for some patients, precluding definitive conclusions regarding long-term survival. Future prospective multi-center studies with standardized eligibility criteria and longer follow-up are warranted to validate these findings and to define the role of NIR-PIT within multidisciplinary treatment strategies for recurrent or metastatic head and neck cancer.

## 5. Conclusions

NIR-PIT was independently associated with improved OS in patients with unresectable recurrent or metastatic head and neck cancer without distant metastasis. These findings are preliminary and hypothesis-generating; therefore, causal inference cannot be established. Nevertheless, the results support the hypothesis that NIR-PIT may contribute to prolonged survival in carefully selected patients and provide a rationale for further investigation, including its integration with systemic therapies. Prospective, multi-center studies are warranted to validate these preliminary observations. Although these results are preliminary, they suggest that NIR-PIT can prolong OS in patients with recurrent/metastatic head and neck cancer without distant metastasis.

## Figures and Tables

**Table 1:** Characteristics of the patients.

Clinical Characteristics	NIR-PIT		Pharmacotherapy		*p*-Value
Median age (range)	68	(50–81)	70	(51–93)	0.657
Sex					0.096
Male	17	(77.3%)	22	(95.7%)	
Female	5	(22.7%)	1	(4.3%)	
ECOG performance status					
0	16	(72.7%)	17	(73.9%)	
1	6	(27.3%)	5	(21.7%)	
2	0	(0.0%)	1	(4.3%)	
Primary site					0.832
Oral cavity	5	(22.7%)	7	(30.4%)	
Oropharynx	7	(31.8%)	4	(17.4%)	
p16-positive	1	(4.5%)	1	(4.3%)	
p16-negative	6	(27.3%)	3	(13.1%)	
Nasopharynx	3	(13.6%)	1	(4.3%)	
Hypopharynx	2	(9.0%)	5	(21.7%)	
Larynx	1	(4.5%)	2	(8.7%)	
Sinonasal sinus	3	(13.6%)	4	(17.4%)	
Salivary gland	1	(4.5%)	0	(0.0%)	
rT category					0.200
0, 1, 2	11	(50.0%)	10	(43.5%)	
3, 4	11	(50.0%)	13	(56.5%)	
rN category					0.135
0	20	(91.0%)	16	(69.6%)	
1, 2, 3	2	(9.0%)	7	(30.4%)	
Target lesion					0.047
Local	20	(90.9%)	16	(69.6%)	
Regional	1	(4.5%)	7	(30.4%)	
Local and Regional	1	(4.5%)	0	(0.0%)	
Carotid artery invasion					0.109
No	22	(100.0%)	19	(82.6%)	
Yes	0	(0.0%)	4	(17.4%)	
Prior treatment for primary site					0.396
Surgery	4	(18.2%)	6	(25.1%)	
Radiotherapy	8	(36.3%)	11	(47.9%)	
Surgery and Radiotherapy	10	(45.5%)	6	(25.1%)	
	median	(range)	median	(range)	
Body mass index (range)	20.2	(16.8–25.6)	19.6	(15.2–27.5)	0.256
Albumin (g/dL)	3.9	(2.9–4.7)	3.9	(2.3–4.4)	0.214
Lactate dehydrogenase (u/L)	179	(141–235)	151	(123–332)	0.169
Neutrophil/lymphocyte ratio	3.28	(1.49–12.7)	5.62	(1.37–21.02)	0.097
Platelet/lymphocyte ratio	229.3	(110.8–784.7)	274.3	(109.6–717.0)	0.602
Modified glasgow Prognostic score					0.197
0	17	(77.3%)	12	(52.2%)	
1	3	(13.6%)	5	(21.7%)	
2	2	(9.1%)	6	(26.1%)	

ECOG: Eastern Cooperative Oncology Group; NIR-PIT, near-infrared photoimmunotherapy.

**Table 2:** BOR, ORR, and DCR.

	NIR-PIT		Pharmacotherapy	
BOR (%)				
CR	7	(31.8%)	1	(4.3%)
PR	11	(50.0%)	8	(34.8%)
SD	2	(9.1%)	8	(34.8%)
PD	2	(9.1%)	6	(26.1%)
ORR (%)	18	(81.8%)	9	(39.1%)
DCR (%)	19	(90.9%)	17	(73.9%)

BOR, best overall response; ORR, objective response rate; DCR, disease control rate; CR, complete response; PR, partial response; SD, stable disease; PD, progressive disease; NIR-PIT, near-infrared photoimmunotherapy.

**Table 3:** Adverse events.

Adverse Event	Any Grade *n*	(%)	Grade 1 *n*	Grade 2 *n*	Grade 3 *n*	Grade 4 n	%Grade 3–4
Pain	22	(100)	7	15	0	0	0
Laryngeal edema	13	(59)	5	6	1	1	9
Drug-induced pneumonitis	1	(4.5)	0	1	0	0	0
Fistula	1	(4.5)	0	0	1	0	4.5
Sepsis	1	(4.5)	0	0	0	1	4.5
Photosensitivity	0	(0)	0	0	0	0	0

**Table 4:** Univariate analysis for factors associated with OS in patients eligible for NIR-PIT.

			Overall Survival		
Parameter	Category	*n*	HR	(95%CI)	*p*-Value
Age	<75	23	27	(14-NA)	0.338
	≧75	9	8	(1-NA)	
Sex	Male	26	17	(11.5-NA)	0.152
	Female	6	35	(18.5-NA)	
ECOG performance status	0	23	27	(14-NA)	<0.001
	1	9	16	(2-NA)	
Primary site	Oral	8	16	(1–21)	<0.001
	Pharynx	15	NA	(11.5-NA)	
	Larynx	2	6.5	(2-NA)	
	Sinonasal sinus	6	15.5	(5-NA)	
	Salivary gland	1	28	(NA-NA)	
Target lesion	Local	26	27	(14-NA)	0.209
	Regional	5	8	(1-NA)	
	Local and Regional	1	21	(NA-NA)	
Longest diameter of the target lesion	<30 mm	17	27	(11-NA)	0.935
	≧30 mm	15	18.5	(12.5-NA)	
Treatment modality	Pharmacotherapy	10	8	(1–25)	0.003
	NIR-PIT	22	35	(16-NA)	
Disease control rate	CR, PR, SD	28	28	(16-NA)	0.01
	PD	4	13.3	(2-NA)	
Body mass index	<18.5	8	17.3	(2–27)	0.271
	≧18.5	24	28	(12.5-NA)	
Albumin (g/dL)	<1.4	17	17	(8-NA)	0.342
	≧1.4	15	27	(18.5-NA)	
Neutrophil/lymphocyte ratio	<5.2	22	28	(11.5-NA)	0.486
	≧5.2	10	18.5	(2-NA)	
Platelet/lymphocyte ratio	<120	2	11	(1-NA)	0.11
	≧120	30	27	(14-NA)	
Modified Glasgow Prognostic score	0	22	27	(11.5-NA)	0.445
	1, 2	10	17	(1-NA)	

CR, complete response; PR, partial response; SD, stable disease; PD, progressive disease; NIR-PIT, near-infrared photoimmunotherapy; CI, confidence interval; HR, hazard ratio; NA, not available; ECOG, Eastern Cooperative Oncology Group; OS, overall survival.

**Table 5:** Multivariate analysis for factors associated with OS in patients eligible for NIR-PIT.

	Overall Survival		
Parameter	HR	(95%CI)	*p*-Value
ECOG Performance Status	2.68	(0.87–8.31)	0.087
Primary site	0.65	(0.35–1.22)	0.18
Treatment modality (NIR-PIT, Pharmacotherapy)	0.24	(0.07–0.78)	0.018
Disease control rate (CR PR SD, PD)	7.81	(1.22–50.05)	0.03

CR, complete response; PR, partial response; SD, stable disease; PD, progressive disease; NIR-PIT, near-infrared photoimmunotherapy; CI, confidence interval; HR, hazard ratio; ECOG, Eastern Cooperative Oncology Group; OS, overall survival.

**Table 6:** Association of post-treatment changes in NLR and PLR with overall survival.

	Overall Survival	
Parameter	HR (95%CI)	*p*-Value
ΔNeutrophil-to-lymphocyte ratio (baseline − post-treatment)	0.813 (0.662–0.999)	0.049
ΔPlatelet-to-lymphocyte ratio (baseline − post-treatment)	0.995 (0.989–1.001)	0.083

Association of post-treatment changes in NLR and PLR with overall survival: Univariate Cox models. ΔNLR and ΔPLR were defined as baseline minus post-treatment values. Post-treatment values were measured immediately prior to the subsequent treatment session (i.e., the next scheduled therapy). Therefore, a larger Δ indicates a greater post-treatment decrease in the respective index. NLR, neutrophil-to-lymphocyte ratio; PLR, platelet-to-lymphocyte ratio; HR, hazard ratio; CI, confidence interval.

## Data Availability

Data supporting the results of this study are available from the corresponding author upon reasonable request.

## Supplementary Materials

The following supporting information can be downloaded at: https://www.mdpi.com/article/10.3390/cancers18030350/s1. Table S1: Univariate analysis for factors associated with OS in the entire cohort of patients. Table S2: Multivariate analysis for factors associated with OS in the entire cohort of patients. Figure S1: Kaplan-Meier curves of OS in patients in the entire cohort of patients.

## Abbreviations

The following abbreviations are used in this manuscript:

BOR	best overall response
CI	confidence interval
CR	complete Response
CRP	c-reactive protein
DCR	disease control rate
ECOG PS	Eastern Cooperative Oncology Group performance status
EGFR	epidermal growth factor receptor
HR	hazard ratio
ICIs	immune checkpoint inhibitors
mGPS	modified Glasgow Prognostic Score
NIR-PIT	near-infrared photoimmunotherapy
NLR	neutrophil-to-lymphocyte ratio
ORR	objective response rate
OS	overall survival
PD	progressive disease
PD-1	programmed cell death protein 1
PDT	photodynamic therapy
PLR	platelet-to-lymphocyte ratio
PR	partial response
QOL	quality of life
RECIST	response Evaluation Criteria In Solid Tumors
SCC	squamous cell carcinoma
SD	stable disease
