# Supplementary material for: Outcomes of Near-Infrared Photoimmunotherapy for Head and Neck Cancer: A Single-Center Retrospective Study
Source: Cancers (Basel). 2026 Jan 23;18(3):350. doi: 10.3390/cancers18030350 (PMC12896801; doi:10.3390/cancers18030350)

Supplementary Appendix

Table of Contents

Table S1. Univariate analysis for factors associated with OS in the entire group of patients.
Table S2. Multivariate analysis for factors associated with OS in the entire group of patients.
Figure S1. Kaplan-Meier curves of OS in patients in the entire group of patients.

OS was significantly prolonged in the NIR-PIT group compared with the pharmacotherapy group. Median OS was 35 months in the NIR-PIT group and 8 months in the systemic pharmacotherapy group (log-rank p = 0.001). Median follow-up was 40 months in the NIR-PIT group and 49 months in the Pharmacotherapy group

Table S1.

Univariate analysis for factors associated with OS in the entire cohort of patients.

			Overall Survival		
Parameter	Category	n	HR	(95%CI)	*p*-value
Age	<75	34	18.5	(12-35)	0.529
	≧75	11	8	(2-33)	
Sex	Male	39	16	(10-27)	0.078
	Female	6	35	(18.5-NA)	
ECOG performance status	0	33	25	(12.5-35)	0.111
	1, 2	12	9.5	(3-28)	
Primary site	Oral	12	12	(4-21)	0.165
	Pharynx	22	33	(12-NA)	
	Larynx	3	11	(2-NA)	
	Sinonasal sinus	7	14	(5-NA)	
	Salivary gland	1	28	(NA-NA)	
Target lesion	Local	36	18.5	(12-35)	0.328
	Regional	8	8	(1-NA)	
	Local and Regional	1	21	(NA-NA)	
Longest diameter of the target lesion	< 30mm	22	25	(8-NA)	0.785

	≧ 30mm	23	17	(10-35)	
Carotid artery invasion	No	41	18.5	(11.5-28)	0.675
	Yes	4	22.5	(6-NA)	
Treatment modality	non-NIR-PIT	23	8	(4-25)	0.001
	NIR-PIT	22	35	(16-NA)	
Disease control rate	CR, PR, SD	37	27	(12-NA)	<0.001
	PD	8	6	(2-14)	
Body mass index	<18.5	12	10	(2-27)	0.0569
	≧18.5	33	25	(12.5-NA)	
Albumin (g/dL)	<1.4	25	12.5	(6-28)	0.0858
	≧1.4	20	27	(18.5-NA)	
Lactate dehydrogenase (u/L)	<124	1	27	(NA-NA)	0.954
	≧124	44	18.5	(11.5-33)	
Neutrophil/lymphocyte ratio	<5.2	28	28	(12-NA)	0.0269
	≧5.2	17	10	(4-27)	
Platelet/lymphocyte ratio	<120	3	4	(1-NA)	0.0317
	≧120	42	18.5	(12-33)	
Modified Glasgow Prognostic score	0	29	27	(16-NA)	0.0472
	1, 2	16	12	(6-17)	

Table S2.

Multivariate analysis for factors associated with OS in the entire cohort of patients.

	Overall Survival		
Parameter	HR	(95%CI)	*p*-value
Neutrophil/lymphocyte ratio	2.54	(1.02-6.32)	0.046
Platelet/lymphocyte ratio	0.08	(0.02-0.34)	<0.001
Modified Glasgow Prognostic score	0.95	(0.32-2.87)	0.93
Treatment modality (NIR-PIT, Pharmacotherapy)	0.26	(0.11-0.64)	0.003
Disease control rate (CR PR SD, PD)	5.18	(1.52-17.65)	0.008

Figure S1.

Kaplan-Meier curves of OS in patients in the entire cohort of patients.

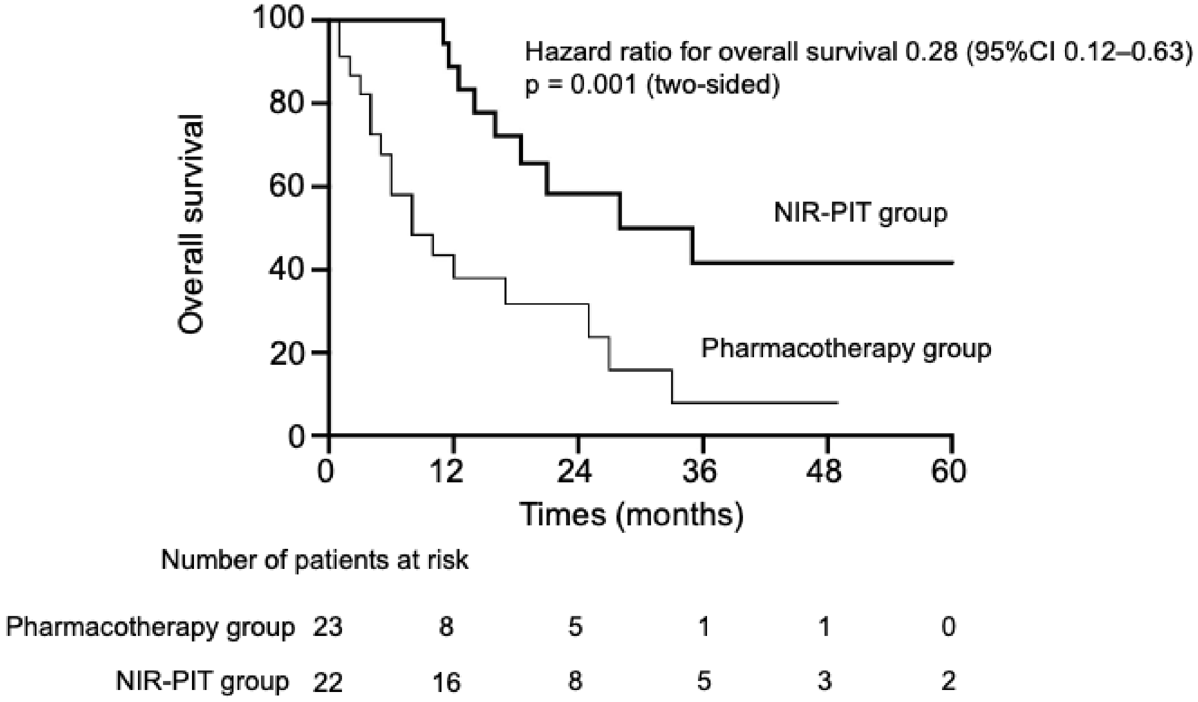

Supplement: Supplementary file 1 [file cancers-18-00350-s001.zip › cancers-4046333-supplementary.pdf]
